# Comparison of MTA and CEM Cement Microleakage in Repairing Furcal Perforation, an In Vitro Study

**Published:** 2013-03

**Authors:** S Sahebi, F Moazami, N Sadat Shojaee, MK Layeghneghad

**Affiliations:** a **Dept. of Endodontics, School of Dentistry, Shiraz University of Medical Science, Shiraz, Iran**; b **Dentist**

**Keywords:** MTA, CEM, Microleakage, Fluid Filtration Method

## Abstract

**Statement of Problem:** Sealing the perforation defect is an important factor to reduce inflammation in the area and to perform healing. Selecting the appropriate material to repair the defect is an important concern. Among the various available materials, MTA and CEM are used recently for achieving this purpose. In the current study we compare the sealing ability of these materials by evaluating their microleakage by fluid filtration method.

**Purpose:** The purpose of this study was to compare the microleakage of MTA and CEM cement in furcal perforation in different periods of time.

**Materials and Method:** Forty one mandibular molars were selected for this experimental study. The perforation defects were created perpendicular to the long axis of the teeth, on the furcation of the teeth and the samples were divided into 2 experimental and two control groups. The defects were sealed by CEM and MTA in each experimental group. The samples were undergone the fluid filtration test with 20 cm H2O pressure. The amount of fluid filtration was measured for each sample at 24, 72 and 168 hrs and the data were analyzed by using ANOVA and T test.

**Results:** The experimental groups which were sealed with CEM exhibited significantly less microleakage in all determined periods of time (24, 72 and 168 hrs) than MTA groups (p< 0.001).

**Conclusion:** Based on the results of this study, CEM cement has a better sealing ability compared with MTA using fluid filtration method.

## Introduction

Perforations are procedural accidents that occur during endodontic treatment and affect the long term prognosis of the tooth. The prognosis of the endodontic treatment is affected by the size, location, and time of perforation and also the ability of the material used to seal the defect [1].

To minimize the contamination of perforation area, it is important to provide an adequate seal immediately [2]. Various materials have been used to repair the perforation and there are some criteria suggested for the ideal repairing material. These criteria include biocompatibility, sealing ability, noncytotoxicity and the ability to induce osteogenesis and cementogenesis [3].

MTA was introduced in 1993 by Lee and Torabinejad for repair of lateral root perforations [4]. These cements composed of dicalcium silicate, tricalcium silicate, tricalcium aluminate and tetracalcium aluminoferite [5]. MTA is a biocompatible repairing material and has many clinical applications. This material is used in the treatment of open apex teeth, pulpotomy, pulp capping, root end filling and management of perforation [6-10] and pulp revascularization [11]. Comparing to other restorative materials, MTA is suggested as a superior material to repair furcal perforations [6, 8, 12].

Despite all of these appropriate properties, this cement also has some shortcomings.MTA has a long setting time (four hours) and its pH value is 12.5 [13]. Also gray MTA can cause color changes which have been reduced in the white MTA by lessening the iron component [14]. Poor handling [15] and relatively high price are other objections of MTA.

Recently, a novel endodontic cement, consisted of calcium compounds (i.e. calcium oxide, calcium carbonate, calcium silicate, calcium sulfate, calcium hydroxide, calcium chloride) has been introduced to endodontics [16-17].

The clinical application of this cement is similar to MTA; however it has better results than MTA when used as a pulp capping agent [18-19] or in pulpotomy of permanent molars with irreversible pulpitis and treatment of internal root resorption [19]. CEM was also used as a root end filling material and showed acceptable results in comparison with MTA [20-21]. CEM cement has antibacterial effects better than MTA and comparable with calcium hydroxide [22] and similar to MTA, CEM has low cytotoxicity on different cell lines [23-24].

 Several studies evaluated the ability of MTA to seal furcal defects and it appeared to be an appropriate material for repairing the furcal perforations [6, 8 and 12]. Samaie et al evaluated the histological response to MTA and CEM in furcal perforation area and they showed similar favorable biological responses for both materials [25]. 

The ability of restorative material to seal the perforation defect in vitro, has been evaluated by different methods included bacterial leakage model [26], radioisotopes [27], dye penetration [4], and fluid filtration method [28]. We chose the fluid filtration technique for leakage assessment, because it would permit a quantitative measurement of microleakage over a longitudinal time period without destruction of the experimental specimens [29].To date there has been a lot of studies compared the sealing ability of these two materials with other methods except fluid filtration. The purpose of this study is to compare the microleakage of MTA and CEM cement in repairing the furcal perforation employing fluid filtration method.

## Materials and Method

Forty two extracted human mandibular molars with closed apex and completely distinct roots were selected. Teeth with cracks, carries or resorptions were excluded from the study.

The teeth were stored in NaOCl (Sedr Sehat, Iran) 0.5% for 48 hours to be disinfected and tissue remnants were removed. Then, the teeth were kept and stored in the normal saline solution (NaCl 0.9%, Darupakhsh, Iran) during the study. 

The occlusal surface of the crowns and 5 mm of the apical portion of the roots were cut using high speed diamond disc (D&Z, Darmstadt, Germany) with water coolant. Pulp tissues and remnants were removed by stainless steel Barbed Broache (Dentsply Maillefer, Japan) and were irrigated with normal saline, then the whole canal from orifices to apical portion were sealed by composite (Tetric N-bond Ivoclar Viva dent, Germany).

According to Chau study the perforation defect was created by using Stander’s system with drill number size 1.5 mm in furcal area [30]. All samples were observed by the microscope (Zeiss, Germany) under ×6 magnification to determine the cracked teeth. One of the teeth was cracked and excluded from the study. 

The samples were randomly divided into 2 experimental groups with 15 and 16 teeth and two control groups with 5 teeth. After irrigation with normal saline ProRoot MTA, (tooth colored formula, Dentsply, Tulsa, OK, USA) and CEM cement (Bionique Dent, Tehran, Iran) were prepared according to their manual instructions. Both materials were applied into the perforation site and compacted with the moist cotton pellets. Then the teeth were placed in room temperature, each tooth was covered in wet gauze to provide 100% humidity and stored in a closed jar for 24 hours to allow the repair materials completely being set.

The perforations were made but were not sealed in the positive control group. Perforation defect was not created, for the negative control group.

**Figure1a:**
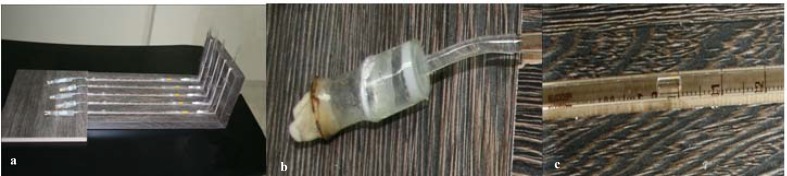
Fluid filtration device b A plastic tube was attached to the crowns of the teeth c Air bubble was displaced in the scaled pipette

Microleakage was measured by using the fluid filtration method as described by Hardy et al [29]. A device was designed to measure the microleakage (Figure 1a). A plastic tube attached to the crowns of the teeth with a cianoacrylate adhesive (Figure 1b). A scaled pipette (HBG, Germany) in which, each unit was equal to 0.01 ml, was connected to the tube and filled with water. Then the water in the pipette was removed by pulling the syringe (gauge 27) 2 mm back, to create a small air bubble (Figure 1c). A regular water pressure of 20cm H2O (this pressure was selected to stimulate physiologic condition like the marrow spaces of bone) [31] was forced crown of all samples when the pipette was placed horizontally at 24, 72 and 168 hrs and data were recorded for each sample in experimental and control groups. The displacement of the air bubble was measured by counting the pipette’s lines. Data were analyzed using ANOVA test and then T test.

(Figure 1c). A regular water pressure of 20cm H2O (this pressure was selected to stimulate physiologic condition like the marrow spaces of bone) [31] was forced crown of all samples when the pipette was placed horizontally at 24, 72 and 168 hrs and data were recorded for each sample in experimental and control groups. The displacement of the air bubble was measured by counting the pipette’s lines. Data were analyzed using ANOVA test and then T test.

## Results

In the positive control group, the extent of displaced liquid was 31.22 ml /min which showed complete leakage. In the negative control group, there was no bubble movement. The Means and Standard deviations of the experimental groups in different time periods for MTA and CEM cement were shown in Table 1. The microleakage in MTA group in 24, 72 and 168 hrs was more than CEM cement and the values were significantly different (Table 1). 

**Table 1:** Means and Standard deviations of experimental groups

Time	Group	Means and Standard deviations	N
24 hours	MTA CEM	0.5069 (0.28357) 0.1220 (0.08046)	16 15
72 hours	MTA CEM	0.8750 (0.35920) 0.2260 (0.12094)	16 15
7 days	MTA CEM	1.2512 (0.42343) 0.3127 (0.14533)	16 15

**Figure 2:**
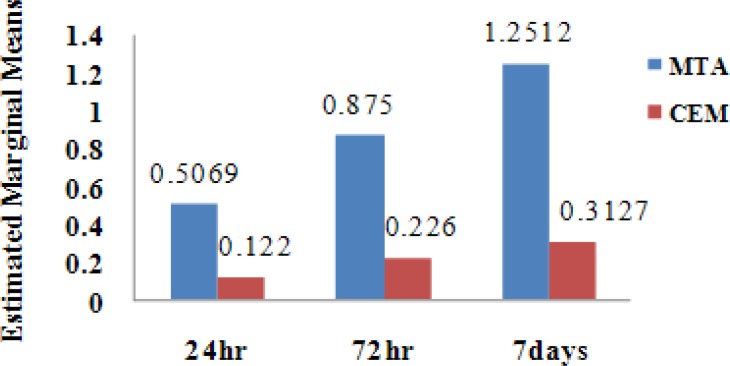
Microleakage in MTA and CEM cement experimental groups in different time periods

Figure 2 shows that MTA groups significantly tend to have more microleakage than CEM cement groups at all determined times (p< 0.001).

## Discussion

MTA is considered as an appropriate material to seal perforations. This endodontic material has many expected criteria like biocompatibility, non-cytotoxicity, radiopacity, availability and tissue regeneration [3]. MTA with pH 12.5 has been shown less microleakage than other repairing materials and it has the ability to induce cementogenesis and osteogenesis [4, 26]. This material is composed of small hydrophilic particles and is compatible with moist conditions like perforated area [32].

CEM cement was introduced to endodontics by Asgary et al in 2006 [16]. This cement was formulated using different calcium compounds. CEM exhibited acceptable film thickness, flowability and reasonable sealing ability compared to MTA [33]. Despite the different compositions, the clinical applications of MTA and CEM are the same. 

Sealing ability of repairing materials can be measured by many different techniques, including bacterial leakage model [26], radioisotopes [27], dye penetration [4], and fluid filtration method.

Some studies have utilized fluid filtration method to evaluate the microleakage of repairing materials [34-36]. This technique is an appropriate method to measure the amount of microleakage over a period of time so it was chosen for leakage assessment in the current study. There are many studies which compare the microleakage of MTA with other restorative materials and they showed MTA had better sealing ability [29, 35-36]. Asgary et al compared the sealing ability of MTA and CEM cement in an invitro study using dye penetration method [37].

Previous studies showed that a bone marrow space pressure was approximately 10-20 mmHg (13.5-27 cm H2O) [31]. We subjected repairing materials to pressures of 20 cm H2O which was approximately similar to that pressure to stimulate physiologic condition like the marrow spaces of bone. 

The results of the present study indicated that MTA leaked more than CEM cement in all scheduled periods. In contrast Asgary et al compared the sealing ability of 3 types of MTA and CEM cement by dye penetration method and found that there was no difference between these materials [37]. Dye penetration method is a popular method because the dyes are available and safe materials, but in this method the amount of microleakage cannot be measured precisely and evaluation of the microleakage in a time period is not possible. Bacterial analysis is also one of the best methods to stimulate clinical conditions. Kazem et al showed CEM cement, Root MTA and White MTA have similar microleakage using dye and bacterial penetration method [38]. In another in vitro study Yavari et al compared polymicrobial microleakage of some endodontic materials. They showed MTA and CEM had more sealing ability than amalgam and composite resin [39]. Perhaps, the different methodologies which were used in these studies explain the different results. The fluid filtration method measures the amount of microleakage in a period of time. Considering the superiority of the fluid filtration method for precise evaluation of quantities, the current study showed the microleakage of CEM was less than the MTA.

CEM cement has good handling properties and is not sticky, so it did not adhere to applicator and is condensed easily. This material has slight expansion due to its composition, calcium sulfate and calcium silicate and is hydrated continuously after initial setting and then further crystalline maturation is occurring [37]. In an in vitro study Hasheminia et al concluded that CEM cement was superior sealing ability compared with MTA in saliva contaminated condition [40]. 

Also in an in vitro study Ghorbani et al compared the microleakage of CEM in two different media. They concluded that CEM cement could seal the perforations more effectively with PBS than stilled water [41]. These characteristics of CEM cement may explain the superior sealing ability of this material compared with MTA.

As the results of in vitro tests may not show the full clinical potential of the tested material to seal perforation defects, we suggest future invivo researches to evaluate the sealing ability of MTA and CEM cement.

## Conclusion

The result of the present study showed that CEM cement had less microleakage compared with MTA in all periods of times using fluid filtration method and it may be a good alternative for MTA.
